# A new rapid method for detecting epidermal growth factor receptor mutations in non-small cell lung cancer

**DOI:** 10.3892/or.2015.3716

**Published:** 2015-01-14

**Authors:** MIYAKO TAKATA, HIROKI CHIKUMI, KEIJI MATSUNAMI, MASAHIRO KODANI, TOMOHIRO SAKAMOTO, KAZUHIRO HASHIMOTO, MASAKI NAKAMOTO, KENSAKU OKADA, TSUYOSHI KITAURA, SHINGO MATSUMOTO, JUN KURAI, AKIRA YAMASAKI, TADASHI IGISHI, NAOTO BURIOKA, EIJI SHIMIZU

**Affiliations:** 1 Division of Medical Oncology and Molecular Respirology, Department of Multidisciplinary Internal Medicine, Tottori University, Yonago-shi, Tottori-ken, Japan; 2 Center for Infectious Diseases, Tottori University Hospital, Yonago-shi, Tottori-ken, Japan; 3 Trust Medical Co., Ltd., Hyogo, Japan; 4 Exploratory Oncology Research and Clinical Trial Center, National Cancer Center, Kashiwa, Chiba, Japan; 5 Department of Pathobiological Science and Technology, School of Health Science, Tottori University, Yonago-shi, Tottori-ken, Japan

**Keywords:** ultrarapid PCR, EGFR mutations, non-small cell lung cancer, epidermal growth factor receptor

## Abstract

Mutations in the epidermal growth factor receptor (*EGFR*) gene are associated with a favorable clinical response to the EGFR tyrosine kinase inhibitors gefitinib and erlotinib in non-small cell lung cancer (NSCLC). We present here, a new method for the rapid detection of the two most common *EGFR* mutations (delE746-A750 and L858R) from clinical samples. The methodology involves the combination of newly designed mutation-specific primers and a novel real-time PCR machine with an innovative thermo-control mechanism that enables ultrarapid PCR. We evaluated this method using a cell mixture composed of various ratios of lung cancer cells harboring mutated or wild-type *EGFR*, lung cancer tissues obtained by surgery, and a cytology sample obtained by bronchoscopy from a lung cancer patient. In the cell mixture analysis, our method detected 0.1% of cells with delE746-A750 and 1% of cells with L858R among cells with wild-type *EGFR*. In 143 lung cancer tissues, the result of this assay was concordant with those of direct sequencing in 138 samples. The five samples with discordant results were tested using a PCR-Invader assay and the result matched those of our method at 100%. We also successfully detected *EGFR* mutations in the lavage obtained from a lung cancer patient. The turnaround time for this method was <10 min, and all steps could be accomplished in <50 min after sample collection. Thus, our novel PCR method offers a rapid, simple, and less expensive test for *EGFR* mutations and can be applied as a point-of-care diagnostic test.

## Introduction

Lung cancer is the leading cause of death from cancer globally (1). Non-small cell lung cancer (NSCLC) accounts for more than 80% of all lung cancer cases. Few NSCLC patients are diagnosed at an early stage and patients with advanced disease are treated with platinum-based combination chemotherapy; however, the objective response rate is very low (2). Resent advances in understanding the molecular basis of lung cancer has led to practical implementation of epidermal growth factor receptor (EGFR)-targeted treatment. The EGFR tyrosine kinase inhibitor (TKI) gefitinib was approved for the treatment of NSCLC in Japan in January 2002, and activating somatic mutations in *EGFR*, conferring sensitivity to EGFR TKIs were discovered in 2004 (3). Since then, EGFR TKIs, such as gefitinib and the equally effective erlotinib, have become the first-line treatment option for NSCLC patients in which the tumor harbors activating *EGFR* mutations, based on the results of a number of phase III trials (4–9). Therefore, in modern clinical settings, *EGFR* mutation testing has become essential for offering the most suitable therapy for a patient with advanced NSCLC.

The historical standard for *EGFR* mutation testing has been direct sequencing of DNA extracted from samples of resected tumor or from biopsies. This method is advantageous as it can be applied to discover ‘new’ mutations; to date, nearly 30 mutations in exons 18–21 have been detected in lung cancer specimens (3,10–14). However, direct sequencing has several limitations. The method requires complex steps and a few days to obtain a result. More importantly, the sensitivity of this method is low; mutant DNA must comprise ~20% of all the DNA in a sample in order to be reliably detected (15). Therefore, when the diagnosis is based on cytology samples that contain a very low percentage of tumor cells, direct sequencing is not applicable.

More recently, based on findings that the most common *EGFR* mutations are a 15-bp in-frame deletion in exon 19 (delE746-A750) and a point mutation in exon 21 (L858R), which together account for ~90% of cases with *EGFR* mutations (16), more focused and mutation-specific approaches have been developed. These methods, PCR-Invader (17,18), peptide nucleic acid-locked nucleic acid (PNA-LNA) PCR clamp (19), cycleave PCR (20), and Scorpion Amplification Refractory Mutation System (ARMS) (21) are PCR-based methods that can detect known *EGFR* mutations with higher sensitivity and a shorter turnaround time than direct sequencing. Therefore, these methods are now frequently used in modern clinical laboratory practice.

However, these methods still have several limitations. These methods adopt relatively complex PCR technologies with pre-designed fluorogenic probes, are packaged by manufacturers, and are often available through outside reference laboratories at relatively expensive rates. The turnaround time for receiving results is 3–5 days, which can sometimes create a bottleneck for immediately starting TKI therapy in patients. Moreover, the cost of the testing renders repeated examination impossible; yet, this may sometimes be required for patients in whom the disease recurs after prior TKI therapy. Therefore, more rapid and less expensive *EGFR* mutation testing is required.

Here, we developed a new, simple, PCR-based method for the detection of the two most common *EGFR* mutations. This assay involves a pair of mutation-specific primers used in combination with a newly developed PCR machine that is equipped with a novel thermo-control mechanism that makes ultrarapid PCR cycling possible. In the present study, we evaluated this approach for *EGFR* mutation detection in tumor tissue gathered during resection and showed the feasibility of using this approach in a cytology sample collected by bronchoscopic examination.

## Materials and methods

### Cell lines and DNA samples

All lung cancer cell lines used in the present study originated from adenocarcinoma. The 11–18 cell line was obtained from the Cell Resource Center for Biomedical Research (Tohoku University, Sendai, Japan). The Ma1 cell line was provided by Dr Hirashima (Osaka Prefectural Habikino Hospital, Osaka, Japan). The A549 cell line was purchased from the American Type Culture Collection (Rockville, MD, USA). The *EGFR* mutation status of these cell lines was examined in our previous study (22). Cells were maintained in DMEM (Wako, Osaka, Japan) supplemented with 10% fetal bovine serum (Life Technologies, Carlsbad, CA, USA), 50 U/ml penicillin, and 50 U/ml streptomycin (both from Wako). Genomic DNA was prepared using a Wizard^®^ Genomic DNA Purification kit (A1120; Promega, Madison, WI, USA) according to the manufacturer’s instructions.

### Clinical samples

Ethical approval was obtained from the Tottori University Hospital and fully informed written consent was obtained from all patients involved prior to the surgery or tissue collection.

Tumor tissues were obtained from surgical specimens of resected tumors, from 143 lung cancer patients treated at Tottori University Hospital; these samples were embedded in Tissue-Tek OCT Compound (Sakura Finetechnical, Tokyo, Japan), and were immediately frozen at −80°C. Macrodissection of the OCT-embedded tissue samples was performed to enrich the final proportion of the tumor DNA, and DNA was extracted using the Wizard^®^ Genomic DNA Purification kit. For samples with discordant results between direct sequencing and mutation-specific PCR, a PCR-Invader method was performed by BML, Inc. (Tokyo, Japan) as a reference test.

### Direct sequence analysis

For direct sequence analysis exon 19 and 21 of *EGFR*, the following PCR primers were used: *EGFR* exon 19F, 5′-GCAATATCAGCCTTAGGTGCGGCTC-3′ and *EGFR* exon 19R, 5′-CATAGAAAGTGAACATTTAGGAT GTG-3′; and *EGFR* exon 21F, 5′-CTAACGTTCGCCAGCC ATAAGTCC-3′ and *EGFR* exon 21R, 5′-GCTGCGAGCTCA CCCAGAATGTCTGG-3′. The PCR conditions were as follows: 1 cycle at 94°C for 9 min, followed by 40 cycles each consisting of 94°C for 1 min, 57°C for 1 min, and 72°C for 2 min, and a final cycle at 72°C for 5 min. The PCR products were purified with a MultiScreen-PCR filter plate (Millipore, Tokyo, Japan) and then sequenced using a BigDye Terminator v3.1 cycle sequencing kit and an ABI PRISM 3130xl genetic analyzer (Applied Biosystems, Foster City, CA, USA).

### Design of mutation-specific PCR primer sets

We designed a deletion-specific primer for the delE746-A750 mutation within exon 19 and a point mutation-specific primer for the L858R mutation within exon 21 of *EGFR*. Sequences of the primer sets were as follows: PCR forward primer for delE746-A750, 5′-CACAATTGCCAGTTAACGTCTTC-3′ (19DF) and PCR reverse primer for delE746-A750, 5′-TGTTGGCTTTCGGAG ATGTTTTG-3′ (19DR3); PCR forward primer for L858R, 5′-TCCCATGATGATCTGTCCCT-3′ (21F2f) and PCR reverse primer for L858R, 5′-CACCCAGCAGTTTGGTCC-3′ (21ARMS3).

### Mutation-specific PCR using a conventional thermal cycler

For conventional PCR amplification using the mutation-specific primer sets, PCR conditions were as follows: the reaction mixtures contained 2 μl of 10X PCR buffer, 0.5 μl of dNTPs, 1 μl of each allelic-specific primer (10 μM), 0.2 μl of AmpliTaq^®^ Gold DNA polymerase (Applied Biosystems), 1 μl of template DNA, and 14.3 μl of ddH_2_O in a total volume of 20 μl. Thermal cycling conditions on a PCR Thermal Cycler Dice (Takara, Shiga, Japan) were as follows for the delE746-A750 mutation: 1 cycle at 94°C for 9 min, followed by 35 cycles each consisting of 94°C for 1 min, 59°C for 1 min, and 72°C for 2 min, and a final cycle at 72°C for 5 min. Similarly, for the L858R mutation, conditions involved 1 cycle at 94°C for 9 min, followed by 35 cycles at 94°C for 1 min, 64°C for 1 min, and 72°C for 2 min, and a final cycle at 72°C for 5 min. The PCR products were then electrophoresed on agarose gels and stained with ethidium bromide.

### Mutation-specific PCR using an ultrarapid PCR machine

**Figure 1:**
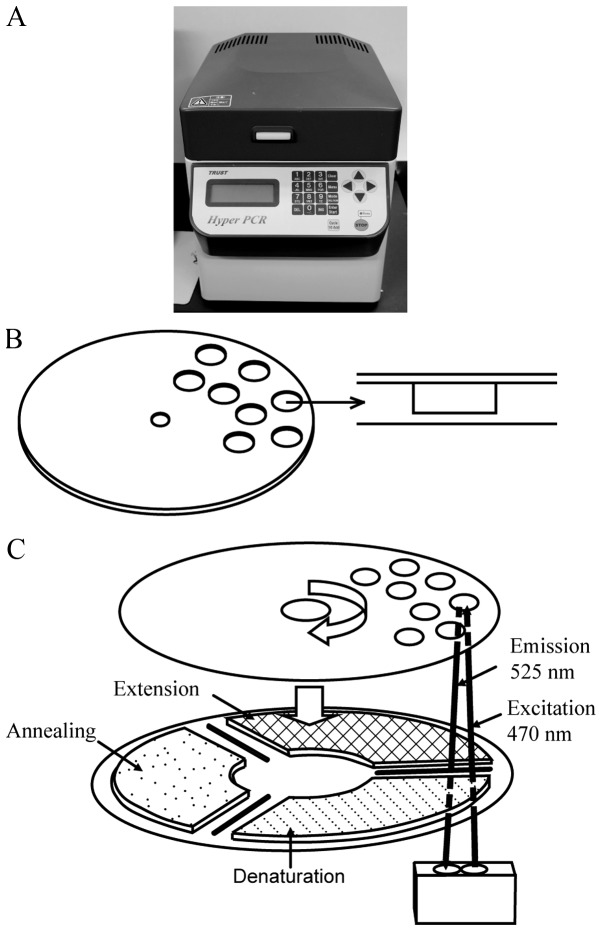
Architecture of the ultrarapid real-time PCR, UR104MK III. (A) External appearance of the machine. (B) A PCR reaction mixture is pipetted onto a flat well in a plastic disk, and sealed with thin film. (C) By high-speed rotation of the disk onto 3 independently controlled thermo-elements, ultrarapid PCR can be accomplished in <10 min. Fluorescence occurring with the production of PCR products is automatically monitored during each cycle, and immediately after ultrarapid PCR, melting curve analysis can be performed to verify product purity.

For ultrarapid PCR-based *EGFR* mutation detection, we utilized a newly developed high-speed real-time PCR machine, termed the ‘Hyper-PCR’ UR104MK III (Trust Medical, Hyogo, Japan), which was jointly developed by ourselves and Trust Medical (23). The UR104MK III employs a novel temperature control technology. In this system, the PCR mixture is enclosed in a small vessel on a thin, flexible plastic disk and sealed with adhesive film, and the disk is rotated rapidly onto three separated heat elements. By controlling the speed of rotation and the temperature of the three heat elements, rapid PCR can be accomplished. Real-time monitoring of the fluorescent dsDNA dye produced during PCR progression, and the ability to perform melting curve analysis of the PCR product are also incorporated into this machine (Fig. 1). The typical time for amplification and detection when using this apparatus is <10 min.

The optimized reaction mixtures for use with this machine contained 1.6 μl of 10X Fast Buffer I, 1.3 μl of a 2.5 mM dNTP mixture, 0.4 μl of each allele-specific primer (10 μM), 0.2 μl of SpeedSTAR HS DNA Polymerase (5 U/μl) (Takara), 1 μl of template DNA, 1.6 μl of 1:2,000 SYBR^®^-Green I nucleic acid gel stain (Cambrex Biosciences, Rockland, ME, USA), and 9.5 μl of ddH_2_O in a total volume of 16 μl. Furthermore, dimethyl sulfoxide (DMSO) Hybri-Max^®^ (Sigma, St. Louis, MO, USA) was added to a final concentration of 5%. Thermal cycling conditions for ultrarapid PCR were as follows for the delE746-A750 mutation: 1 cycle at 94°C for 1 min, followed by 35 cycles each including 98°C for 1.30 sec, 55°C for 5.00 sec, and 72°C for 3.00 sec. Similarly, for the L858R mutation, conditions entailed 1 cycle at 94°C for 1 min, followed by 30 cycles each consisting of 98°C for 1.30 sec, 68°C for 8.00 sec, and a further 68°C for 8.00 sec. Total PCR cycling time for the delE746-A750 and the L858R mutation detection was within 6 and 9 min, respectively. Following PCR cycling, melting curve analysis was performed within 4 min.

For interpretation of the ultrarapid PCR results, criteria used in other studies of qualitative real-time PCR analysis were applied (24). In brief, to be considered as a positive result, a fluorescence signal generated during ultrarapid PCR should display an exponential amplification above the threshold level and the obvious crossing point (Cp) (25), with a single peak upon melting curve analysis, giving a unique melting temperature (Tm) value. A signal was considered as negative when no Cp value was obtained within the amplification cycles.

## Results

### Establishment of EGFR mutation-specific ultrarapid PCR

**Figure 2:**
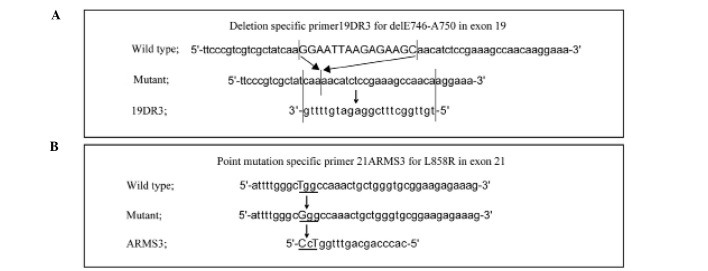
Mutation-specific primers. (A) The reverse primer specific for delE746-A750 in exon 19 (19DR3) was designed to anneal only to the genome containing a deletion of nucleotides 2235–2249, by connecting the regions flanking the deleted sequence. (B) The reverse primer specific for L858R in exon 21 (ARMS3) was designed to be homologous to nucleotides 2573–2590 of the mutant allele. An additional mismatch (C to T substitution) at the -2 position from the 3′-end of the primer was introduced to improve discriminatory ability. The forward primer for each mutation was designed to be homologous to the stable area of each exon.

We first established a specific PCR to detect mutations within exons 19 and 21 of *EGFR*, which are representative mutations underlying the responsiveness of NSCLC to EGFR inhibitors (3). The genomic sequence of *EGFR* was retrieved from the NCBI database (NM_005228). For the in-frame deletion within exon 19, which removes nucleotides 2235–2249, causing a deletion of amino acids 746 through 750 (delE746-A750), we designed a deletion-specific primer, 19DR3 (Fig. 2A). This primer was designed to anneal only to the genomic sequence harboring the nucleotide 2235–2249 deletion, by connecting the flanking sequences on either side of the deletion. By shortening the 3′-end of the primer that corresponded to the upstream genomic sequence, we could improve the specificity of the primer.

For the exon 21 amino acid substitution, in which G is substituted for T at nucleotide 2573, causing an amino acid substitution of L to R (L858R), a point mutation-specific primer, 21ARMS3, was designed (Fig. 2B). Here, we employed the ARMS technique, and designed the primers to be refractory to PCR amplification of non-matching target sequences (26,27), by also including an additional mismatch in the candidate point mutation-specific primers (ARMS1-10) at positions −2 or −3 from the 3′-end of the primers (data not shown). Among these candidate primers, we chose the primer (ARMS3) that allowed discrimination without decreasing the sensitivity and specificity of amplification in a series of experiments in which these candidate primers were tested under the same temperature and time conditions in ultrarapid PCR.

The forward primers for each mutation-specific primer were designed to match the stable area of each *EGFR* (19F and 21F). The concentration of the PCR primers and magnesium, the annealing temperature and other cycling parameters, and the type of DNA polymerase used were determined by exploration, and the conditions described here are the final optimized conditions.

### Specificity of the mutation-specific primers

**Figure 3:**
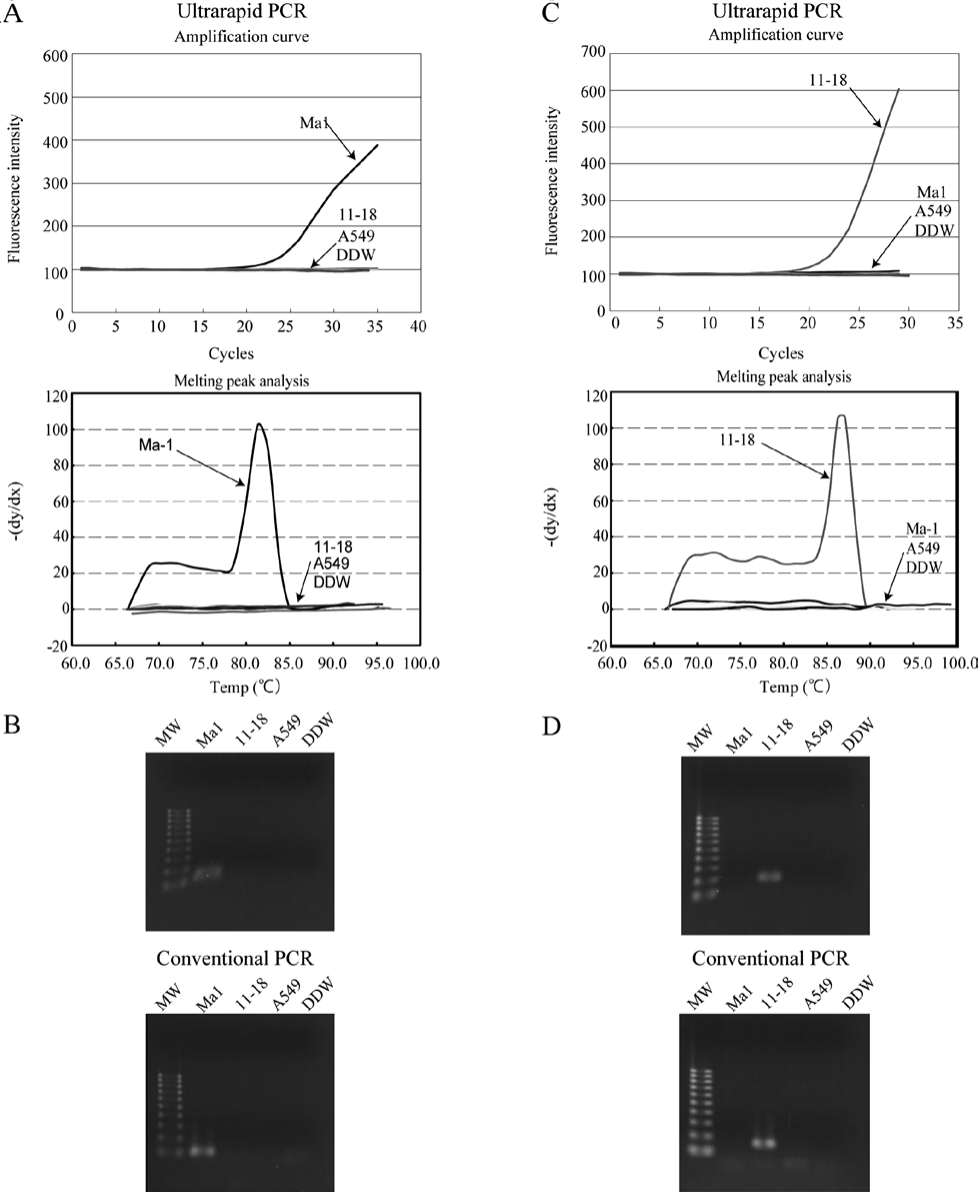
Specificity of the mutation-specific primers for the two most frequent *EGFR* mutations. (A) The delE746-A750-specific primers were tested on Ma1 cells harboring delE746-A750, 11–18 cells harboring L858R, A549 cells with the wild-type *EGFR*, and a negative control [double-distilled water, (DDW)] using ultrarapid PCR. The amplification and melting curve, and electrophoresed amplified product is shown. MW, 100 bp-DNA ladder. (B) The experiment was also performed using conventional PCR, and the results of electrophoresis of the corresponding products are shown. (C) The L858R-specific primers were tested on each cells and negative control using ultrarapid PCR. (D) The L858R-specific primers were tested on the same samples using conventional PCR, and the results of electrophoresis of the products are shown. EGFR, epidermal growth factor receptor.

To evaluate the specificity of the mutation-specific primers, ultrarapid PCR using the mutation-specific primers was performed on lung cancer cell lines, and the concordance of these results with those of conventional PCR was evaluated. *EGFR* genotyping of the lung cancer cell lines Ma1, 11–18 and A549 had been performed by sequence analysis in our previous study, and were revealed as delE746-A750, L858R and wild-type, respectively (22). As shown in Fig. 3A, using primer sets, 19DF and 19DR3, a significant increase in fluorescence intensity was observed upon ultrarapid PCR only in Ma1 cells that harbor delE746-A750, and melting curve analysis revealed a clear peak at the expected Tm (81.3°C). The PCR product was visualized by agarose gel electrophoresis and the expected product of 113 bp was detected. To validate the data, conventional PCR was performed using the same primer sets, and a product of the same size was detected only in Ma1 cells (Fig. 3B).

A similar experiment was performed using the L858R point mutation-specific primers (21F2f and 21ARMS3) in ultrarapid PCR. As shown in Fig. 3C, a significant increase in fluorescence intensity and a clear melting curve peak at the expected Tm (87.1°C) was observed only in 11–18 cells that harbor the exon 21 L858R point mutation, and the size of the amplicon was confirmed as 166 bp by agarose gel electrophoresis. The same result was obtained by conventional PCR using this primer set (Fig. 3D). These data revealed that the combination of mutation-specific primers and ultrarapid PCR yielded satisfactory discrimination of the mutant alleles.

### Sensitivity of mutation-specific PCR

**Figure 4:**
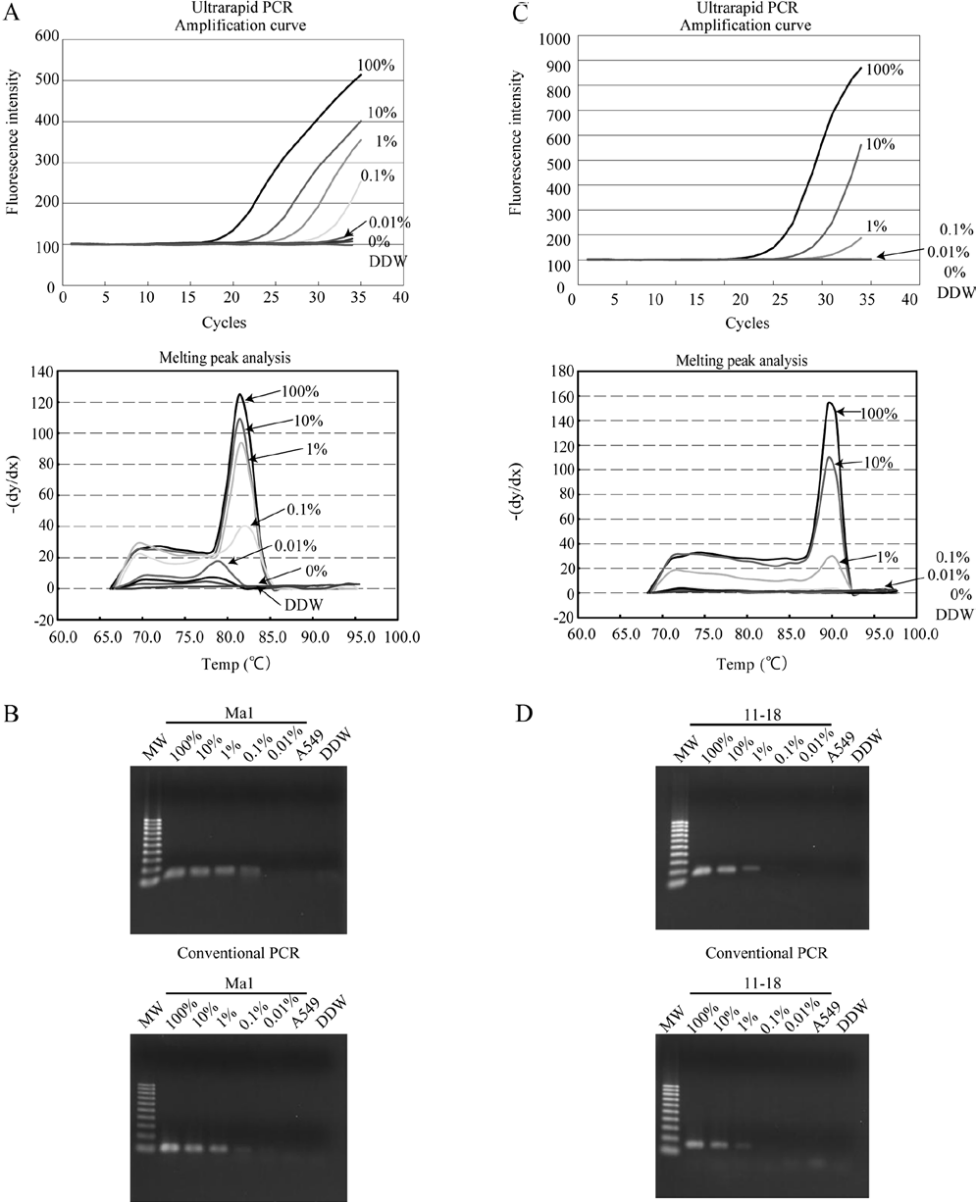
Sensitivity of mutation-specific ultrarapid PCR. (A) Ultrarapid PCR amplification of the delE746-A750 allele from a mixture of cell samples containing 100, 10, 1, 0.1, 0.01 and 0% of Ma1 cells harboring the mutation. As few as 0.1% of tumor cells with the delE746-A750 mutation could be detected. DDW, double-distilled water. (B) Conventional PCR amplification of the same samples as in A. Electrophoresis of the products showed the same detection limit as ultrarapid PCR. (C) Ultrarapid PCR amplification of the L858R allele from a mixture of cell samples containing 100, 10, 1, 0.1, 0.01 and 0% of 11–18 cells harboring the mutation. As few as 1% of tumor cells with the L858R mutation could be detected. (D) Conventional PCR amplification of the same samples as in C; electrophoresed products are shown. The same detection limit as with ultrarapid PCR was observed.

To evaluate the sensitivity of the assay, a serial dilution of lung cancer cell lines carrying *EGFR* mutations (Ma1 or 11–18) into wild-type cell lines (A549) was analyzed by ultrarapid PCR using the mutation-specific primers. Using the delE746-A750 primers in ultrarapid PCR, a mixture containing 0.1% Ma1 cells could be detected as positive from the amplification curve and melting curve analysis, and this result was confirmed by gel electrophoresis (Fig. 4A). The same result was also obtained by conventional PCR (Fig. 4B). Using the L858R primer sets, a cell mixture containing 1% 11–18 cells was detected as positive by ultrarapid PCR, and the result was confirmed by gel electrophoresis of the PCR product (Fig. 4C). The same detection limit was also observed with this primer set with conventional PCR (Fig. 4D). These data revealed that ultrarapid PCR combined with delE746-A750 and L858R mutation-specific primers allowed detection of 0.1 or 1% mutation-carrying lung cancer cells among wild-type cells, respectively.

### Clinical sample testing

**Figure 5:**
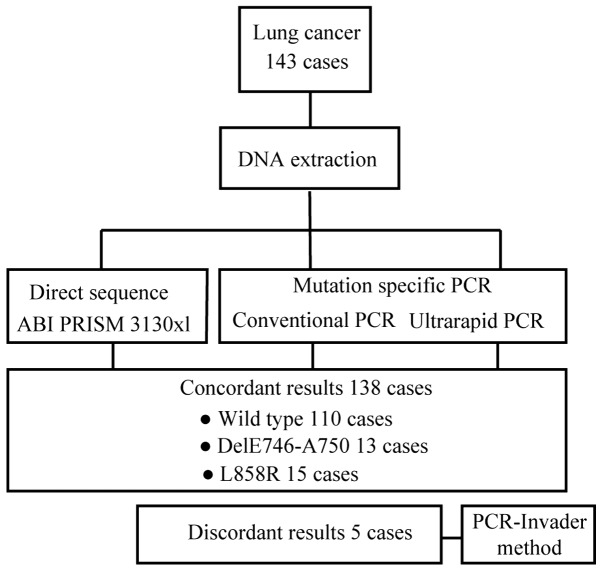
Detection of two major *EGFR* mutations in lung cancer samples. Tumor samples obtained during resection were tested for the presence of two major *EGFR* mutations using mutation-specific primers in ultrarapid PCR or conventional PCR. Direct sequencing was used as comparison, and PCR-Invader was used as a reflex test for samples with discordant results. EGFR, epidermal growth factor receptor.

We evaluated the concordance of the results obtained by direct sequencing and using mutation-specific primers in ultrarapid PCR or conventional PCR in 143 lung cancer tumors. Overall, the results of 138 of 143 samples were concordant among direct sequencing, ultrarapid and conventional PCR (Fig. 5). The remaining five samples that demonstrated inconsistent results are shown in Table I. In these samples, direct sequencing could not detect any mutations, whereas the results with ultrarapid PCR and conventional PCR were concordant: two delE746-A750 and three L858R mutations were identified. To evaluate these discrepant samples, we used PCR-Invader analysis, which confirmed the PCR-based results. Therefore, we concluded that the indicated mutations were indeed present in these samples, yet could not be detected by direct sequencing, possibly due to the lower sensitivity of detection of direct sequencing methodology.

When compared with the concluded *EGFR* mutation status, the sensitivity and specificity of direct sequencing was 84.8 and 100% respectively, whereas those of ultrarapid PCR were 100 and 100%, respectively (Table II). These data revealed that ultrarapid PCR has superior sensitivity and specificity in clinical samples to direct sequencing, and parallels that of the PCR-Invader method, which is one of the commonly used PCR-based methodologies in current clinical practice.

### Ultrarapid detection of EGFR mutation in a patient with adenocarcinoma

**Figure 6:**
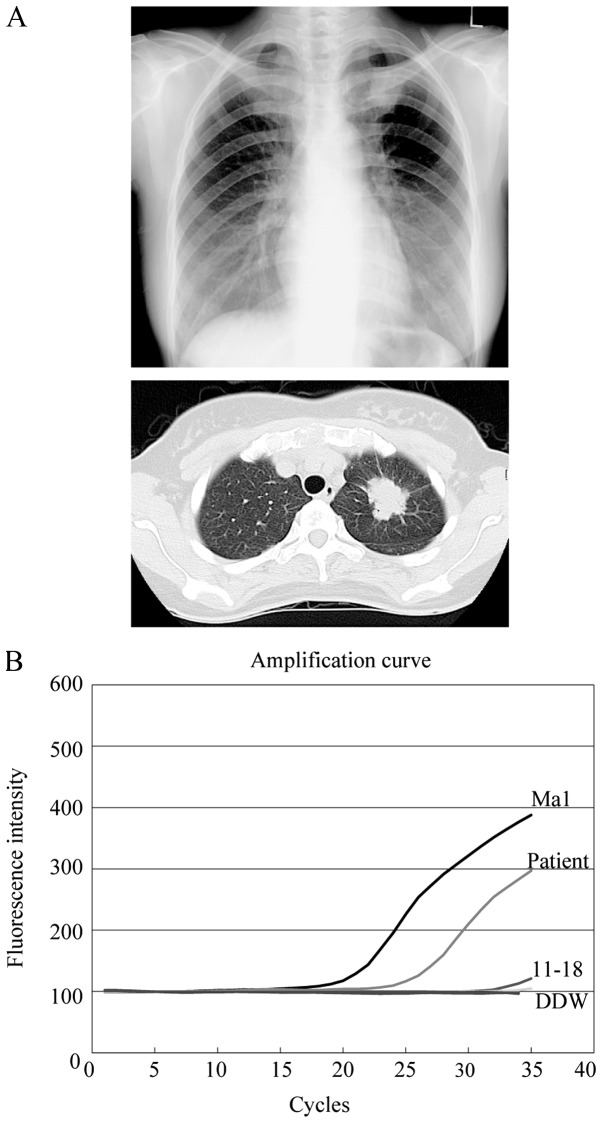
Mutation detection on a cytology sample obtained from a lung cancer patient by bronchoscopic examination. (A) Chest X-ray and computed tomography scan of the chest of a patient referred to our hospital on suspicion of lung cancer. (B) Results of mutation-specific ultrarapid PCR on a lavage sample collected by bronchoscopic examination. The presence of the delE746-A750 mutation was detected by ultrarapid PCR in <50 min after bronchoscopic examination.

A 39-year-old woman with no history of smoking was referred to our hospital due to the presence of an abnormal shadow in her left upper lung field that was noticed during her regular medical checkup (Fig. 6A, upper panel). A computed tomography (CT) scan of the chest of this patient revealed a spiculated nodular shadow with a 35-mm diameter in the superior division of the left upper lobe (Fig. 6A, lower panel). F-2-deoxy-2-fluoro-D-glucose (FDG)-positron emission tomography revealed multiple lesions with high uptake in her liver and vertebrae. Based on the suspicion of adenocarcinoma with multiple metastases, we preformed flexible bronchoscopic examination with washing, brushing and forceps biopsy. In addition to the cytological and histological examination of the lavage and biopsy specimens, we applied ultrarapid PCR analysis to the lavage sample. The lavage was centrifuged and DNA was extracted within 40 min after sample collection. Ultrarapid PCR analysis subsequently revealed the presence of the delE746-A750 *EGFR* mutation in her lavage sample, within 6 min (Fig. 6B). After waiting for confirmation of positive results by cytology and histology, which were obtained 3–4 days after bronchoscopic examination, the patient was diagnosed as having adenocarcinoma with an *EGFR* mutation (cT2aN0M1b, stage IV). She commenced treatment with 150 mg erlotinib/day immediately, and her lung CT scan at 6 weeks after the initiation of treatment revealed a marked improvement.

## Discussion

In the present study, we newly developed an ultrarapid PCR approach for detecting *EGFR* mutations. This method showed excellent specificity and sensitivity for clinical samples, which is superior to direct sequencing and is comparable to other PCR-based methodologies that are frequently used in modern laboratory practice. In addition, to our knowledge, this method has superior rapidity among the methodologies reported for *EGFR* mutation detection. Therefore, introduction of this technology to clinical practice may open new opportunities for diagnosis and therapy of lung cancer patients.

In the present study, we used direct sequencing as a method against which to compare our newly developed ultrarapid mutation-specific PCR, since it is the historical standard and many studies reporting novel methodology have used it for comparison (28). However, even when using macrodissection to enrich tumor DNA in a sample, the sensitivity of direct sequencing was 84.8%, whereas that of ultrarapid PCR was 100%. In addition, it is noteworthy that the mutation analysis results of the five sequencing-discordant samples were consistent between ultrarapid PCR and PCR-Invader, one of the current laboratory-standard PCR-based assays. In a recent study, these frequently used PCR-based assays, i.e., PCR-Invader, PNA-LNA PCR clamp, Scorpion ARMS and cycleave PCR, could detect mutations in at least 1% of mutant/wild-type allele-admixture samples, and showed equal sensitivity and specificity in clinical specimens (tissue and cytology samples) (29). Based on the detection limit in the admixture analysis and the results of surgical resected tissues in our study, the ultrarapid PCR we present here appears to be comparable to current sensitive laboratory-standard PCR-based methodologies for detecting *EGFR* mutations.

Moreover, compared to the other current PCR-based methods, ultrarapid PCR has several advantages. The first significant advantage is the rapid turnaround time for amplification and detection (<10 min in the experiments reported here). This machine employed a novel thermo-control mechanism, with a thermal ramp rate of up to 20°C/sec; this is the shortest ramp rate among the published ramp rate for thermal cyclers (30). By combining this machine with our newly designed mutation-specific primers, choosing an adequate polymerase, optimizing the composition of the reaction mixture, and adjusting the thermal conditions, we have here developed the fastest real-time detection system for *EGFR* mutation screening to date.

The second advantage of our approach is its simplicity and cost-effectiveness. We used the double-strand DNA-binding dye, SYBR-Green I, for real-time detection of the PCR product, whereas other systems use fluorogenic probes. Since *EGFR* mutation detection is a fundamentally qualitative detection, we believe the simplicity, flexibility, and cost-effectiveness of detection using SYBR-Green I may be sufficient for the purpose. Besides their relative expense, the use of fluorogenic probes complicates modification and optimization of real-time PCR, and primers/probes need to be designed according to specific rules, due to the simultaneous annealing of the primers and probes in real-time PCR (31).

Our system was designed to detect the two major *EGFR* mutations for which the majority of clinical evidence supports the use of EGFR TKIs; it is not able to detect all *EGFR* mutations, similar to other PCR-based systems. However, clinical data supporting the use of EGFR TKIs for less common mutations are emerging, and adaptation of the detection system to these more rare mutations will be required in the near feature. Our system may present a convenient way to do this, since it would only require the design of new primers and adjustment of the PCR conditions.

Our ultrarapid mutation-specific PCR will open new potential applications for the clinical management of lung cancer patients. The representative case we presented in the present study illustrates three important characteristics of this test. First, this test can be applied to cytology samples obtained in routine clinical practice, such as bronchoscopic lavage. Second, since the test result can be obtained within <50 min after sample collection, treatment with TKIs can be started very rapidly. Third, since this system is simple and less expensive, this test may enable clinicians to request repeat tests for *EGFR* mutation for a given patient. Repeated testing for *EGFR* mutations is in demand, particularly for recurrent or metastatic lesions, to allow selection of optimal treatment. Eventually, this test can be used as a point-of-care approach, which has not been available for *EGFR* testing to date.

In conclusion, we developed a simple, rapid, and less expensive *EGFR* mutation detection system. This system has comparable sensitivity and specificity to that of recently developed laboratory-standardized PCR-based methods, and, unlike these methods, offers high speed performance. Given the simplicity of the methodology, this system may help to usher in a new phase in *EGFR* mutation testing for the diagnosis and treatment of lung cancer patients.

## Figures and Tables

**Table I:** Samples showing discordant results between sequencing and PCR detection methods.

Case	Direct sequence	Conventional PCR	Ultrarapid PCR	PCR-Invader method
1	Wild-type	DelE746-A750	DelE746-A750	DelE746-A750
2	Wild-type	DelE746-A750	DelE746-A750	DelE746-A750
3	Wild-type	L858R	L858R	L858R
4	Wild-type	L858R	L858R	L858R
5	Wild-type	L858R	L858R	L858R

**Table II:** Sensitivity and specificity of direct sequencing and mutation-specific ultrarapid PCR.

	Direct sequence	Ultrarapid PCR
Sensitivity	84.8% (28/33)	100% (33/33)
Specificity	100% (110/110)	100% (110/110)
